# Postpartum Postural Orthostatic Tachycardia Syndrome in a Patient with the Joint Hypermobility Syndrome

**DOI:** 10.4061/2009/187543

**Published:** 2009-10-11

**Authors:** Khalil Kanjwal, Beverly Karabin, Yousuf Kanjwal, Blair P. Grubb

**Affiliations:** Division of Cardiology, Department of Medicine, The University of Toledo Medical Center, Health Sciences Campus, Mail Stop 1118, 3000 Arlington Avenue, Toledo, OH 43614, USA

## Abstract

Postural orthostatic tachycardia syndrome (POTS) commonly affects women of childbearing age. We report on a 37-year-old woman who developed symptoms of recurrent syncope in the postpartum period. Her head up tilt test and clinical presentation was consistent with POTS.

## 1. Introduction

Recurrent unexpected syncope may have severe consequences and can result in serious injury, especially in a postpartum period when the maternal syncope can have the disastrous effect on the newborn infant as well as the mother. Postpartum syncope has been reported to result in death due to dropping of the infants [1]. We report on a 37-year-old woman who developed recurrent episodes of syncope in the postpartum period.

## 2. Case

A 37-year-old woman with a past medical history of migraine headache and reflux esophagitis was seen in our autonomic clinic for evaluation of recurrent syncope. In the last nine years, she had experienced episodes of debilitating recurrent syncope. Her first episode occurred six months after she gave birth to her daughter. At that time, she described an episode that occurred while standing at work during which she became “fuzzy”, lightheaded, and subsequently suffered from an episode of syncope with a brief loss of consciousness. She had no convulsive activity during this episode. She continued to experience episodes of lightheadedness without frank syncope. Six years after her first episode the symptoms and the frequency of episodes progressed to the point where she experienced approximately eight episodes of lightheadedness and palpitations over a period of six months, occasionally resulting in syncope. In the interim, multiple neurologists evaluated her. Ultimately, a cardiologist sent her for head up tilt test (HUTT). Upon the assumption of upright posture during the tilt test, her heart rate increased from 86 beats per minute (bpm) (baseline) to 126 bpm within 5 minutes, and her blood pressure dropped from 126 mm Hg (baseline) to 90 mm Hg, associated with symptoms of lightheadedness, palpitations and loss of consciousness. She was initially treated with fludrocortisone, which failed to relieve her symptoms. Midodrine (an alpha agonist) and methylphenidate (noncatecholamine sympathomimetic) were also tried but ineffective. She suffered multiple injuries (including head injuries) during these episodes of syncope, and her quality of life had markedly decreased. During one traumatic syncopal episode, she fell while attending physical therapy. In addition, she also suffered from loss of memory and balance problems.

In our clinic, she presented with a blood pressure of 100/80 sitting and 90/64 standing. Her supine heart rate was 70 beats per minute and immediately upon standing rose to 111 beats per minute. The rest of the physical examination was normal except for the presence of joint hypermobility in the bilateral wrists, thumbs, fingers, elbows, and ankles. After review of her history, physical exam, and the extensive evaluation, the diagnostic impression was autonomic dysfunction and orthostatic intolerance most consistent with new onset postpartum postural orthostatic tachycardia syndrome. Her overall clinical picture of orthostatic intolerance and joint findings were most consistent with joint hypermobility. The initial core management choices here were reasonable; indeed the prior management was essentially exactly as our center would recommend.

 She was commenced on Duloxetine, a serotonin/norepinephrine reuptake inhibitor. During her next clinic visit, she reported marked improvement in symptoms, except for headache and mild cognitive impairment. She was started on Cerefolin to help the memory loss [2].

## 3. Discussion

Multiple cardiovascular adaptations occur during pregnancy, which are essential for successful completion of pregnancy. These include an increase in cardiac output, sodium, and water retention with increase in expansion of blood volume and a reduction in peripheral vascular resistance and diastolic blood pressure [3]. These cardiovascular adaptations continue in the postpartum period and allow for return to a nonpregnant state. These changes in the postpartum period have not been well reported or studied [4]. Our patient developed recurrent episodes of syncope in the postpartum period. Postpartum syncope with significant maternal morbidity and one infant mortality has been reported previously [1]. There have been reports that patients with POTS can have a favorable outcome during pregnancy and in postpartum period [5, 6]. The joint hypermobility syndrome might have predisposed our patient to symptoms of orthostatic intolerance and made her vulnerable especially as she returned to the prepregnant state with gradual loss of estrogen/progesterone, induced fluid retention in the postpartum period.

 In patients with POTS, the main mechanism is the consistent failure of the peripheral vascular system to increase resistance during upright posture. Patients with joint hypermobility syndrome even have more difficulty owing to loss of supporting connective tissues in the vessels. Orthostatic intolerance develops in these patients due to the presence of abnormally elastic connective tissue in the vasculature. This results in an increase in vessel distensibility in response to the augmented hydrostatic pressure that occurs during orthostatic stress. Consequently, there is excessive peripheral venous pooling with a resultant compensatory tachycardia. About 70% of patients with hypermobility syndrome may suffer from some form of orthostatic intolerance due to sympathetic dys-regulation [7]. Patients with POTS can present with syncope without a substantial fall in blood pressure. Increase in cerebrovascular resistance occurring during orthostatic stress can explain loss of consciousness in these patients [8].

 These patients are usually treated with fludrocortisone [9] midodrine [10], methylphenidate [11], and selective serotonin reuptake inhibitors [12, 13]. Combined selective serotonin reuptake inhibitors and selective norepinephrine reuptake inhibitors such as duloxetine are most effective. Duloxetine is postulated to alter serotonin receptor density in brainstem area and thus alter autonomic tone.

In our experience, we have found that use of combined selective serotonin reuptake inhibitors and selective norepinephrine reuptake inhibitors has been more effective in POTS than in neurocardiogenic syncope [14].

## 4. Conclusion

POTS in the postpartum period can have serious consequences; indeed the risks associated with syncope can be life threatening not only to mother but also to the child as well. Early recognition and proper management of these patients are essential to prevent serious morbidity and mortality associated with this clinical entity. Long-term outcomes in patients with postpartum POTS are unknown.
